# Cannabis Use Linked to Altered Functional Connectivity of the Visual Attentional Connectivity in Patients With Psychosis and Controls

**DOI:** 10.1093/schizbullopen/sgaa018

**Published:** 2020-04-20

**Authors:** Musa Basseer Sami, Robert A McCutcheon, Ulrich Ettinger, Steve Williams, Dave Lythgoe, Philip McGuire, Sagnik Bhattacharyya

**Affiliations:** 1 Institute of Psychiatry, Psychology and Neurosciences King’s College London, London, UK; 2 University of Bonn, Bonn, Germany; 3 Centre for Neuroimaging Sciences, King’s College London, London, UK; 4 Institute of Mental Health, University of Nottingham, Nottingham, UK

**Keywords:** resting state, cannabis, psychosis, fMRI, dorsal attention network, visual network

## Abstract

**Background:**

Both chronic cannabis use and psychotic disorders are associated with abnormalities in visual attentional processing. Using functional magnetic resonance imaging (fMRI), we sought to determine whether there would be a difference in functional connectivity in patients and controls with and without a history of cannabis use in the visual and dorsal attention networks.

**Methods:**

Resting-state fMRI data were acquired in patients with early psychosis with (EPC = 29) and without (EPNC = 25); and controls with (HCC = 16) and without (HCNC = 22) cannabis use.

**Results:**

There was a patient effect in both Visual-Dorsal Attention Internetwork (*F*(1,87) = 5.326, *P* = .023) and the Visual Network (*F*(1,87) = 4.044, *P* = .047) and a cannabis effect in the Dorsal Attention Network (*F*(1,87) = 4.773, *P* = .032). These effects were specific to the networks examined with no evidence for significant patient or cannabis effects in other canonical networks. Patients with a history of cannabis use showed increased connectivity in the Dorsal Attention Network (134%, *P* = .019) and Visual Dorsal Attention Internetwork (285%, *P* = .036) compared to non-using controls. In the EPC group connectivity of the Visual Network (ρ = 0.379, *P* = .042) and Visual-Dorsal Attention Internetwork (ρ = 0.421, *P* = .023) correlated with visual hallucinations which were significantly different from EPNC (*P* = .011). Dorsal attention network strength correlated with severity of dependence for cannabis (ρ = 0.215, *P* = .04).

**Conclusion:**

We demonstrate specific cannabis and patient effects in networks associated with visual attentional processing. There is a differential association with hallucinatory symptoms in patients with and without a history of cannabis use. This may indicate that dysconnectivity in these networks serves different roles in the context of cannabis use.

## Introduction

Cannabis use and early psychosis are often comorbid with a large meta-analysis showing regular cannabis use in 30%–40% of cases at onset of psychosis and associated with poorer prognosis.^1,2^ However, the neurobiological substrates underlying the association of psychotic disorders in patients who have used cannabis remain unclear. Both cannabis use and psychosis have been associated with perturbations in functional connectivity in line with the “dysconnectivity hypothesis”.^3,4^ However the nature of such dysconnectivity has not been clarified: in psychosis studies have shown hypoconnectivity,^5–8^ hyperconnectivity^9,10^ and mixed patterns^11–13^; as is the case in cannabis use: (hypoconnectivity,^14,15^ hyperconnectivity^16–19^). Reconciliation of connectivity findings in cannabis and psychosis would require study of both cannabis and psychosis groups together.

One line of investigation would be to look at network connectivity of visual attention processing as this is known to be altered in both cannabis use and psychotic disorders. There is evidence for several such abnormalities in psychosis: impairment of smooth pursuit and antisaccades eye movements^20–24^ as well as abnormalities in motion, contrast detection, reading tasks and face perception.^25–27^ Similarly in cannabis use impairment in contrast sensitivity,^28^ attention to motion processing^29^ and visuo-spatial processing^30,31^ has been demonstrated.

Complementing this is evidence of aberrant functional connectivity in visual and attentional networks in both psychosis and cannabis use. There is a body of evidence showing tasks to elicit abnormalities in visual attentional processing in psychosis,^32–34^ recent work has implicated abnormal visual network activation in schizophrenia^35^; and a history of cannabis use has been shown to be associated with visual and attentional reorganization^36^ and associated with greater connectivity between frontal cognitive control regions and the dorsomedial visual area.^37^

Of note we have shown, in our current sample, alterations in smooth pursuit eye movements (SPEMs) in patients with early psychosis with and without a history of cannabis use.^38^ Patients without a history of cannabis use exhibited deficiency in peak gain not seen in patients with a history of cannabis use. Examination of functional connectivity of the brain networks which underpin visual and attentional processes may thus allow us to determine differences in dynamic functional interactions in patients with and without a history of cannabis use.

Therefore, drawing on the dysconnectivity hypothesis, previous literature as well as our findings in SPEM described above, we hypothesized that there would be a perturbation in functional connectivity in patients with a history of cannabis use specifically in the visual and dorsal attention networks. We expected to see evidence for both a cannabis and psychosis effect on functional connectivity and the network which connects the two (the “visual-dorsal attention internetwork”). We also wished to identify specific dysconnectivity in the EPC group which may help in identifying targets for treatment in this poor-prognosis group.

Furthermore the dysconnectivity hypothesis suggests that visual abnormalities in schizophrenia have shared underpinnings with the false inference seen in the positive symptoms of psychosis.^4^ Both visual processing and our cognitive understanding about the external world rely on the integration of perceptual information in the brain with prior expectations and subsequent prediction to create an external world which is perturbed in the presence of dysconnectivity. Although more intuitive for hallucinations a similar mechanism has been argued for delusions.^4,39^ To examine this we tested whether network strength in visual processing regions would correlate with the positive symptoms of psychosis, particularly in relation to hallucinatory symptoms. We also tested to see if functional connectivity in these networks was associated with eye movements, general functioning and cannabis use.

## Methods

### Sample

The EfCiP (**Ef**fect of **C**annabis **i**n Early **P**sychosis; Stanmore Research Ethics Committee: 17/LO/0577) study was a cross-sectional study of individuals aged 18–38 forming 4 groups: (1) patients with early psychosis (presentation to mental health services within 5 years of psychosis onset) with a history of cannabis use (EPC); (2) patients with early psychosis without a history of cannabis use (EPNC); (3) healthy controls with a history of cannabis use (HCC) and (4) healthy controls without such a history (HCNC). Diagnosis was confirmed using Structured Clinical Interview for DSM-IV for Axis I disorders (SCID).^40^ For full details of recruitment see supplementary material. All “non-cannabis users” (EPNC, HCNC) reported use a few times a year, only once or twice or not at all; whereas all “cannabis users” (EPC, HCC) reported use at least a few times each month, more than weekly or daily (supplementary material).

### Clinical and Biological Measures

On the study day, all participants underwent the Structured Clinical Interview for DSM-IV^40^ for diagnosis, Positive and Negative Syndrome Scale (PANSS)^41^ to assess symptom scores and a standardized battery to assess symptoms, functioning and drug use. See supplementary methods for further details.

In an exploratory fashion we devised a score to capture 5 features of use behavior associated with psychosis outcomes (heaviness, early first use, lifetime abuse, persistence and potency) to give a cumulative score out of 10 for each participant from the information collected in the above measures (named Cannabis Psychosis Score [CPS] for further discussion on how these use-behaviors relate to psychosis see supplementary material).

SPEM data were collected from participants using an eye movement battery developed for psychiatric and neurological disease (EyeLink 1000, SR Research Ltd.). SPEM mean velocity gain scores were calculated at 3 sinusoidal target frequencies (0.2, 0.4, and 0.6 Hz) (supplementary material).

### Image Acquisition and Analysis

T1 weighted images and 5-minute resting-state data were acquired on a 32 channel head coil (Nova Medical) on a General Electric 3-Tesla system. Preprocessing was undertaken using the CONN toolbox (version 18.b) for Statistical Parametric Mapping software (SPM 12 (6906))^42^ running in Matlab R2018a (The Mathworks). Cortical parcellation was undertaken using the Gordon atlas, containing 333 cortical nodes.^43^ Full details for acquisition, preprocessing and parcellation procedures are reported in the supplementary material.

### Statistics

Demographic and clinical data were compared across groups using ANOVA tests for continuous measures and chi-squared tests for categorical data. Follow-up post hoc tests were conducted between groups, as appropriate. To compare clinical parameters between EPC and EPNC groups and cannabis use parameters between HCC groups we undertook 2-sided *t*-tests (preceded by Levene’s test for equality of variances) or chi-squared tests as appropriate. Since data on “days since last joint” were notably skewed median and interquartile range (IQR) are reported for this variable and Mann Whitney U tests were used to test statistical significance between EPC and HCC. We further tested whether motion parameters differed between groups using ANOVA (mean motion, mean global signal change). Statistical significance was set at *P* = .05. Statistical analysis was undertaken in IBM SPSS Statistics version 25 (IBM). Network based statistical analysis is detailed below.

### Network Analysis

#### Canonical Networks.

For each participant, the average network strength was defined as the mean z-transformed Pearson’s correlation coefficient between all nodes in the network of interest (ie, mean edge strength). For each participant we calculated average network strength for 3 predefined networks^43^: dorsal attention network (32 × 32 nodes, 496 edges), visual network (39 × 39 nodes, 741 edges), and the visual-dorsal network internetwork, ie, the connectivity between the 2 distinct networks (39 × 32 nodes, 1248 edges).

We then performed 2×2 MANCOVA with follow-up ANCOVA using network strength for the 3 canonical networks as the outcome of interest and cannabis use and psychosis as the predictor variables covarying for mean motion. In further models we added covariates into the ANCOVA models which we expected might have the potential to confound our findings (Fagerstrom and AUDIT scores to index tobacco and alcohol use respectively). Post hoc, we tested differences between the mean functional connectivity for the 4 groups (EPC, EPNC, HCC, HCNC) corrected for motion and applied Bonferroni correction (ie, by multiplying *P* value by 6). Since the controls who did not use cannabis (HCNC) represent “normal” connectivity and deviation from this indicates abnormal connectivity (dysconnectivity) we report the other groups standardized to the HCNC mean.

To test for specificity of our findings to our networks of interest we undertook similar ANCOVAs for all other Networks parcellated by the Gordon Atlas and report these in the supplementary data.

#### Network Based Statistic.

To further determine if there was a specific subnetwork within the Dorsal Attention Network and Visual Network differing between groups (EPC, EPNC, HCC, HCNC) we used the Network Based Statistic (NBS)^8^ (see supplementary methods for a full description). To determine the direction of group differences we extracted the network strength of this network and adjusted for mean motion, and subsequently covarying for Fagerstrom, AUDIT scores. To determine the optimal primary threshold NBS was performed across 100 thresholds ranging from *F* = 2.00 to *F* = 4.00.

Network visualization was undertaken with BrainNet Viewer 1.63 (https://www.nitrc.org/projects/bnv/).^44^

In exploratory analyses, we tested for correlations between network strength and symptoms using 2-tailed Spearman’s ρ. We examined these correlations separately in each patient group (EPC, EPNC). To distinguish between state and trait measures we used PANSS positive scores (total PANSS positive score and P3: Hallucinatory experiences) for state measures, and presence of positive symptoms from Module B of the SCID (visual hallucinations) for a trait measure (ie, if the symptom had ever been experienced). In networks where a significant correlation was found in one group we tested to see if this was significantly different from the correlation coefficient in the other group using 2-tailed Fisher r-to-z transformation. For completeness the correlation of all positive PANSS/SCID measures with Network Connectivity are reported in the supplementary material. We also tested correlations between network strength and GAF, chlorpromazine equivalents, mean SPEM velocity gain and cannabis use measures.

Finally as a supplementary analysis we tested to see if connectivity was related to patient status (schizophrenia spectrum diagnosis or not; affective disorder diagnosis or not; and currently on/off antipsychotic medication). Since these parameters were the same across both patient groups (EPC, EPNC see Results), this is not relevant to our main analysis and is reported in the supplementary material.

## Results

A total of 103 participants were recruited into the study. One HCC was excluded due to cannabis intoxication, one HCNC was excluded due to a prolactinoma. Six participants (3 EPC, 3 EPNC) were not able to have magnetic resonance imaging (MRI) due to contraindications to MRI scanning. Two participants (EPNC) experienced claustrophobia and were unable to have an MRI scan and one self-terminated the scanning session before resting-state acquisition. Hence, MRI and resting-state data were available for 92 participants: EPC: *n* = 29, EPNC: *n* = 25; HCC: *n* = 16; HCNC: *n* = 22.

One patient under an Early Intervention in Psychosis team had a first psychotic episode aged 12, 20 years before inclusion into the study, and had been asymptomatic off-treatment until re-presenting in their 30 seconds. One HCC suffered from Generalized Anxiety Disorder on no treatment and one HCC had Obsessive Compulsive Disorder 8 years prior to the study and was currently in remission maintained on low dose sertraline (50 mg). Exclusion of these cases in sensitivity analysis had no effect on the main results.

### Demographics and Clinical Measures

Demographic and clinical measures by group are shown in table 1. As expected, patients had increased PANSS scores and decreased GAF compared to controls. Controls had higher IQ score compared to patients. Cannabis using patients had increased Fagerstrom and AUDIT scores indicating increased tobacco and alcohol use compared to EPNC. There was no significant difference between EPC and EPNC in all clinical parameters: total PANSS and all subscales, chlorpromazine equivalents, age at diagnosis and duration since diagnosis, days spent in hospital, proportion with schizophrenia spectrum disorder or affective diagnosis, GAF scores and Global Functioning and Social Functioning subscales (all *P* > .34).

**Table 1.** Data Presented in Cells are Proportions for Discrete Data; Means (SDs) for Continuous Data

	EPC	EPNC	HCC	HCNC	*P*-Value
*n*	29	25	16	22	
Sex	23/29 (79%)	16/25 (64%)	10/16 (63%)	11/22 (50%)	All groups: .183
Age	25.57 (3.89)	26.29 (4.74)	27.11 (5.95)	28.16 (5.29)	All groups: .416
Age at first presentation	23.39 (4.06)	23.29 (5.59)	-	-	EPC vs EPNC: .937
AUDIT	8.79 (5.30)	3.40 (4.92)	7.75 (6.43)	3.59 (2.99)	**All groups: <.001** **EPC vs EPNC: .001** **EPC vs HCNC: .002**
Fagerstrom	2.59 (1.74)	0.64 (1.75)	0.75 (1.74)	0 (1.74)	**All groups: <.001** **EPC vs EPNC: .001** **EPC vs HCC: .006** **EPC vs HCNC: <.001**
Diagnosis			-	-	EPC vs EPNC: .476
Bipolar affective	2 (6.9)	2 (8)			
Brief psychotic	1 (3.4)	2 (8)			
Psychotic depression	1 (3.4)	0			
Psychosis NOS	0	2 (8)			
Schizoaffective	8 (27.6)	6 (24)			
Schizophrenia	11 (37.9)	11 (44)			
Schizophreniform	3 (10.3)	2 (8)			
Substance induced	3 (10.3)	0			
Proportion SSD diagnosis	22/29 (76%)	19/25 (77%)	-	-	EPC vs EPNC: .991
Proportion affective diagnosis	11/29 (38%)	8/25 (32%)	-	-	EPC vs EPNC: .649
PANSS	53.24 (18.49)	53.36 (17.78)	34.56 (5.38)	31.05 (2.13)	**All groups: <.001** **EPC vs HCC: <.001** **EPC vs HCNC: <.001** **EPNC vs HCC: <.001** **EPNC vs HCNC: <.001**
PANSS Hallucinations					**EPC vs HCC: <.001** **EPC vs HCNC: <.001** **EPNC vs HCC: <.001** **EPNC vs HCNC: <.001**
Proportion visual hallucinations history	15/29 (52%)	11/25 (44%)			EPC vs EPNC: .571
GAF	70.24 (8.98)	72.96 (11.40)	89.25 (4.93)	93.32 (2.13)	**All groups: <.001** **EPC vs HCC: <.001** **EPC vs HCNC: <.001** **EPNC vs HCC: <.001** **EPNC vs HCNC: <.001**
CPZ equivalents	189.57 (174.15)	184.86 (176.05)	-	-	EPC vs EPNC: .922
Proportion medicated	19/29 (66%)	17/25 (68%)	-	-	EPC vs EPNC: .847
Intelligence Quotient	100.46 (12.73)	100.21 (14.38)	110.28 (6.93)	110.23 (7.98)	**All groups: .003** EPC vs HCC: .053 **EPC vs HCNC: .042** EPNC vs HCC .053 **EPNC vs HCNC: .043**

*Note*: *P*-values are reported for omnibus tests (chi-squared, ANOVA) for all groups: group-wise comparisons reported as appropriate for post hoc tests (chi-squared, post hoc tests Bonferroni correction). Only significant post hoc tests shown. EPC, Early Psychosis with Cannabis use; EPNC, Early Psychosis without Cannabis use; HCC, Healthy Controls with Cannabis Use; HCNC, Healthy Controls without Cannabis Use; *n*, number of participants; SSD, Schizophrenia Spectrum Disorder; CPZ, Chlorpromazine.

The cannabis using groups had a history of heavy cannabis use with no significant difference in cannabis use parameters: age of first use (EPC: x̅ = 16.07 years [SD 2.51]; HCC: x̅ = 16.00 [SD 2.50]); hours subjectively “stoned” in a day (EPC: x̅ = 5.55 [SD 5.01]; HCC: x̅ = 5.77 [SD 4.41]); days to smoke an eighth of an ounce of cannabis (EPC: x̅ = 9.56 [SD 11.36]; HCC: x̅ = 7.96 [SD 9.28]) and number of joints in a day (EPC: x̅ = 2.76 [SD 1.28]; HCC: x̅ = 2.95 [SD 2.21]) (all *P* > .65). There was a wide variability in days since last joint (EPC: median = 65 [IQR: 1–530]; HCC: median = 7 [IQR: 1–1402]) the difference between groups was not significant (*P* = .637). In the EPC group 12/29 (41.4%) tested positive for THC compared to 7/16 of the HCC groups (43.8%) (*P* = .877). There were significantly more individuals with SCID diagnosis of lifetime cannabis use disorder in the EPC group: 23/29 (79.3%) than the HCC group: 8/16 (50%) (*P* = .04), this was expected due to the psychosis comorbidity.

There were no differences in motion parameters between groups (supplementary table 1).

### Canonical Dorsal Attention and Visual Networks

In dorsal attention and visual networks MANCOVA of all 3 network strengths across groups with mean motion as a covariate revealed trend-level patient and cannabis effects (patient: *F*(3,85) = 2.584, *P* = 0.059) (cannabis: *F*(3,85) = 2.446, *P* = 0.069) but no cannabis × patient interaction effect *F*(3,85) = 0.633, *P* = 0.595). Follow-up ANCOVA of the specific networks revealed (1) significant patient effects in the Visual-Dorsal Attention Internetwork (*F*(1,87) = 5.326, *P* = 0.023); Visual Network (*F*(1,87) = 4.044, *P* = .047); and a trend-level effects in the Dorsal Attention Network (*F*(1,87) = 3.402, *P* = .069); and (2) a cannabis effect in the Dorsal Attention Network (*F*(1,87) = 4.773, *P* = .032) but not Visual (*P* > .5) or Visual-Dorsal Attention Network (*P* = .16). Of note these findings were specific to the Visual and Dorsal Attention Networks with no other patient or cannabis group effect for any of the other canonical networks (*P* ≥ .239 see supplementary material). Results remained significant for the patient effect after addition of AUDIT but not Fagerstrom score in the visual networks (*P* = .045) and visual-dorsal internetwork (*P* = .025). For cannabis effect there was no group effect after addition of Fagerstrom and AUDIT scores as covariates.

Post hoc tests to test group differences showed the EPC groups to have highest connectivity compared to healthy controls in the Dorsal Attention Network (EPC mean connectivity strength 134% of HCNC, 95% CI 119%–149%, *P* = .019, Bonferroni corrected) and Visual Dorsal Attention Internetwork (EPC mean connectivity strength 285% of HCNC, 95% CI 196%–369%, *P* = .036, Bonferroni corrected). Group differences can be seen in figure 1. This difference remained significant after addition of Fagerstrom and AUDIT scores in the Visual Dorsal Attention Internetwork (EPC mean connectivity strength 339% of HCNC, 95% CI 230%–507%, *P* = .045, Bonferroni corrected) but not in the Dorsal Attention Network.

**Fig. 1.**
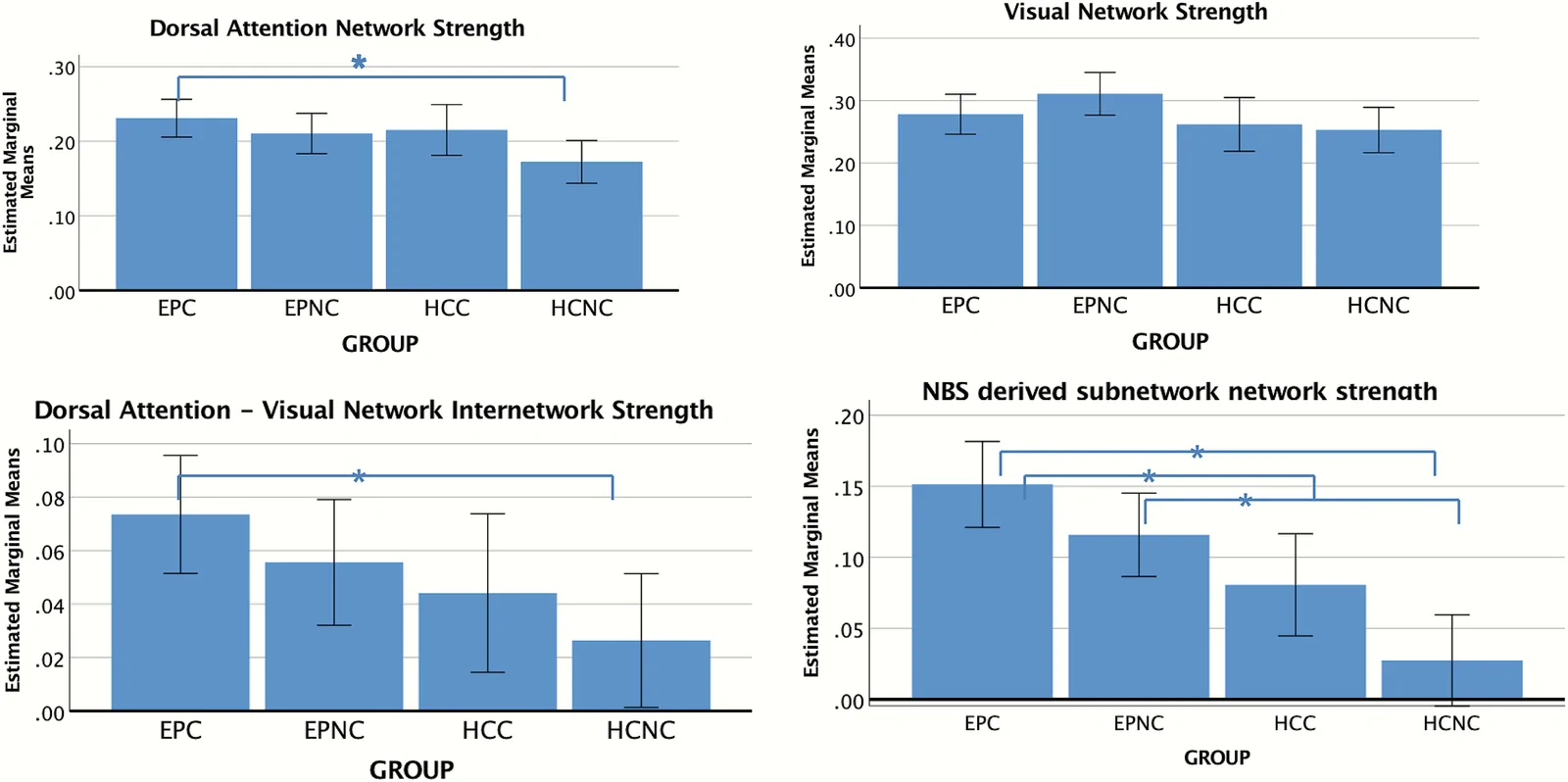
Visual and dorsal attention network strength by group. EPC, Patients with history of cannabis use; EPNC, Patients without history of cannabis use; HCC, Controls with history of cannabis use; HCNC, Controls without history of cannabis use; *Statistically Significant (*P* value < .05; Error bars 95% CIs). Y-axis represents network strength means co-varied for mean motion.

There were no patient or cannabis effects for all other Canonical Networks parcellated by the Gordon Atlas indicating specificity of our findings to our networks of interest (supplementary material).

### Network Based Statistic Defined Network

Significant networks were identified across a range of F thresholds (supplementary material). The most stringent F-Statistic threshold was extracted for further analysis. This network involved almost all nodes of the networks (64/71, 90.1% of all nodes) and 151 edges (151/4970, 3.1% of all possible edges) (figure 2). The direction of connectivity strengths of the NBS defined network was: EPC>EPNC>HCC>HCNC (figure 1). Group differences in network strength remained significant after adjustment for AUDIT and Fagerstrom scores (*P* ≤ .014).

**Fig. 2.**
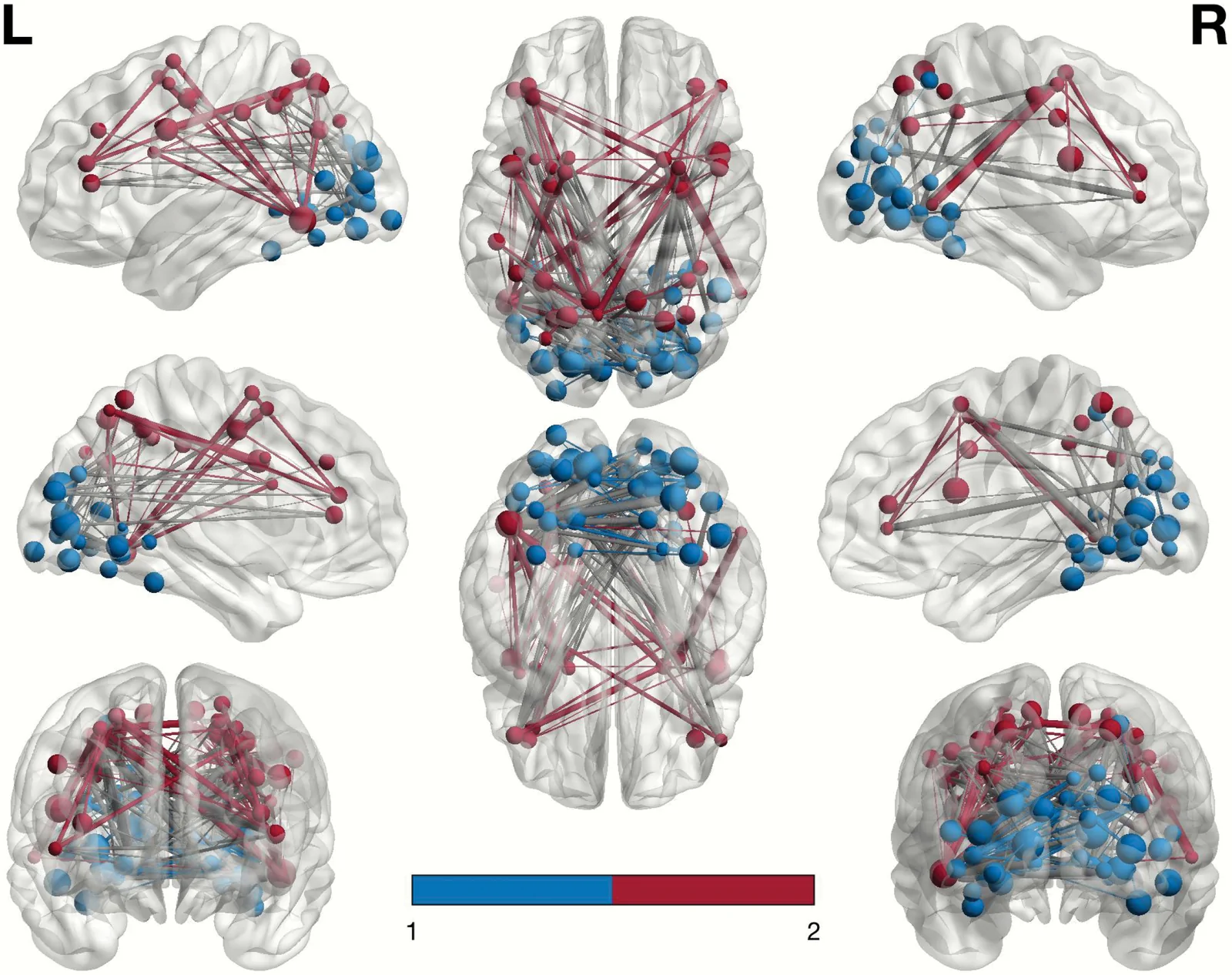


### Correlation of Network Strength With Symptoms

#### Trait Measures.

In the EPC group a history of visual hallucinations positively correlated with network strength in the following networks: visual network (ρ = 0.379, *P* = .042), visual-dorsal attention internetwork (ρ = 0.421, *P* = .023) and NBS defined network (ρ = 0.487, *P* = .007). No such correlation existed in the EPNC group. The difference in correlations for EPC and EPNC were statistically significant (visual network (*P* = .011); visual-dorsal internetwork (*P* = .011) and NBS defined network (*P* = .014)).

#### State Measures.

In the EPNC group PANSS rated hallucinatory behavior inversely correlated with network strength in the visual network (ρ = −0.499, *P* = .011) whereas no such correlation was evident in the EPC group. The difference in correlations for EPC and EPNC was statistically significant (*P* = .0031). In the EPNC group the visual network (ρ = −0.589, *P* = .002), dorsal attention network (ρ = −0.416, *P* = .038) and NBS defined network (ρ = −0.499, *P* = .011) inversely correlated with PANSS positive scores whereas no such correlation was evident in the EPC group (figure 3). The difference in correlations for EPC and EPNC were statistically significant: for the visual network (*P* = .007) and NBS defined network (*P* = .05).

**Fig. 3.**
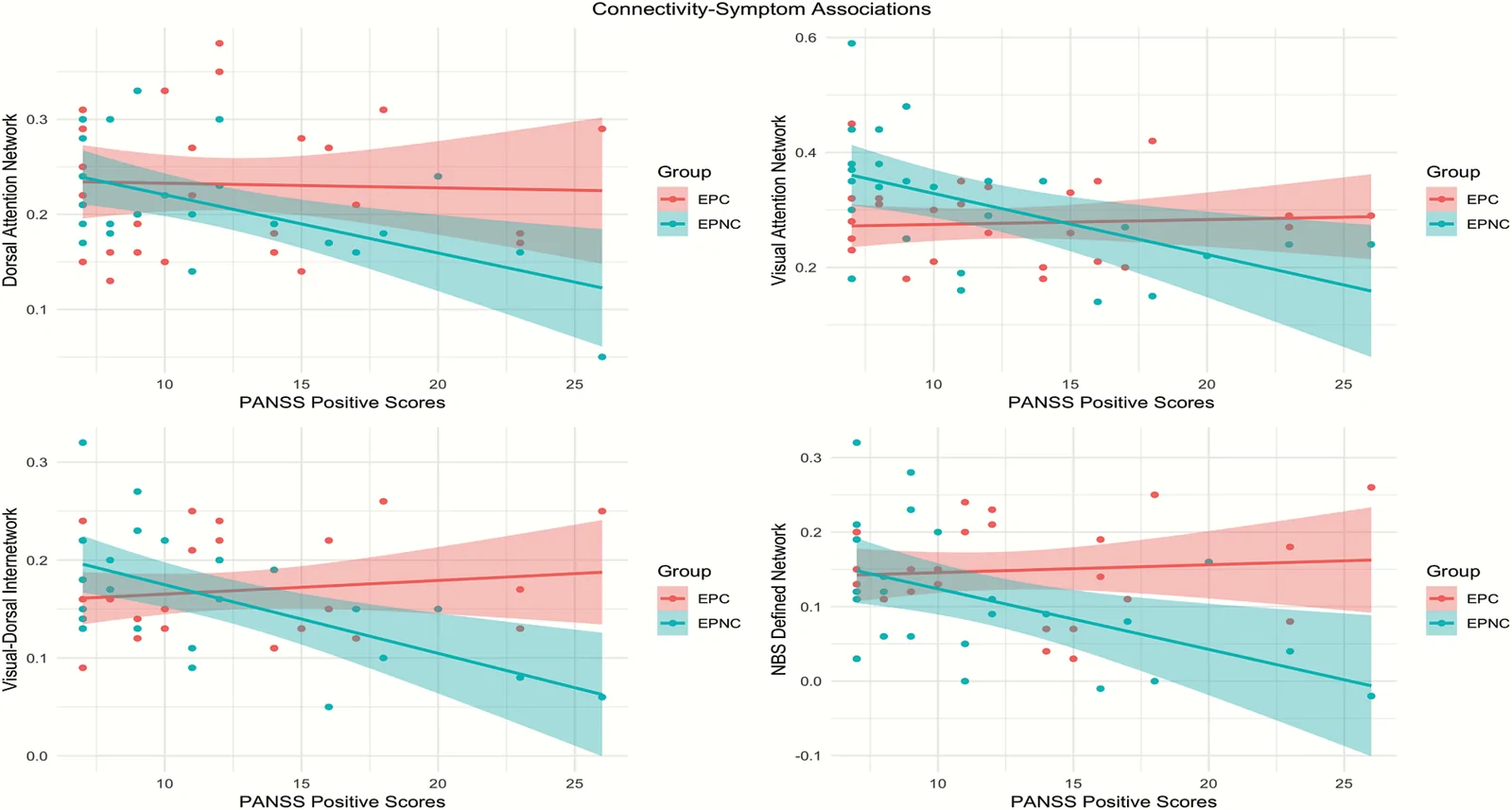
Network strength—PANSS symptom correlation by patient group. EPC, Patients with history of cannabis use; EPNC, Patients without history of cannabis use. Error bars represent 95% Confidence Intervals.

#### Cannabis Use.

Dorsal Attention Network strength positively correlated with Severity of Dependence Scale for cannabis use (ρ = 0.215, *P* = .04) and for the CPS score (ρ = 0.281, *P* = .007). The CPS score further positively correlated with internetwork strength (ρ = 0.266, *P* = .011), and NBS defined network strength (ρ = 0.350, *P* < .001). To ensure this association was not accounted for by group difference between users and non users we further tested correlation restricted to the cannabis using groups (EPC and HCC). CPS score was associated with Dorsal Attention Network strength (ρ = 0.424, *P* = .004), internetwork strength (ρ = 0.369, *P* = .014) and NBS defined network strength (ρ = 0.298, *P* = .05) (figure 4).

**Fig. 4.**
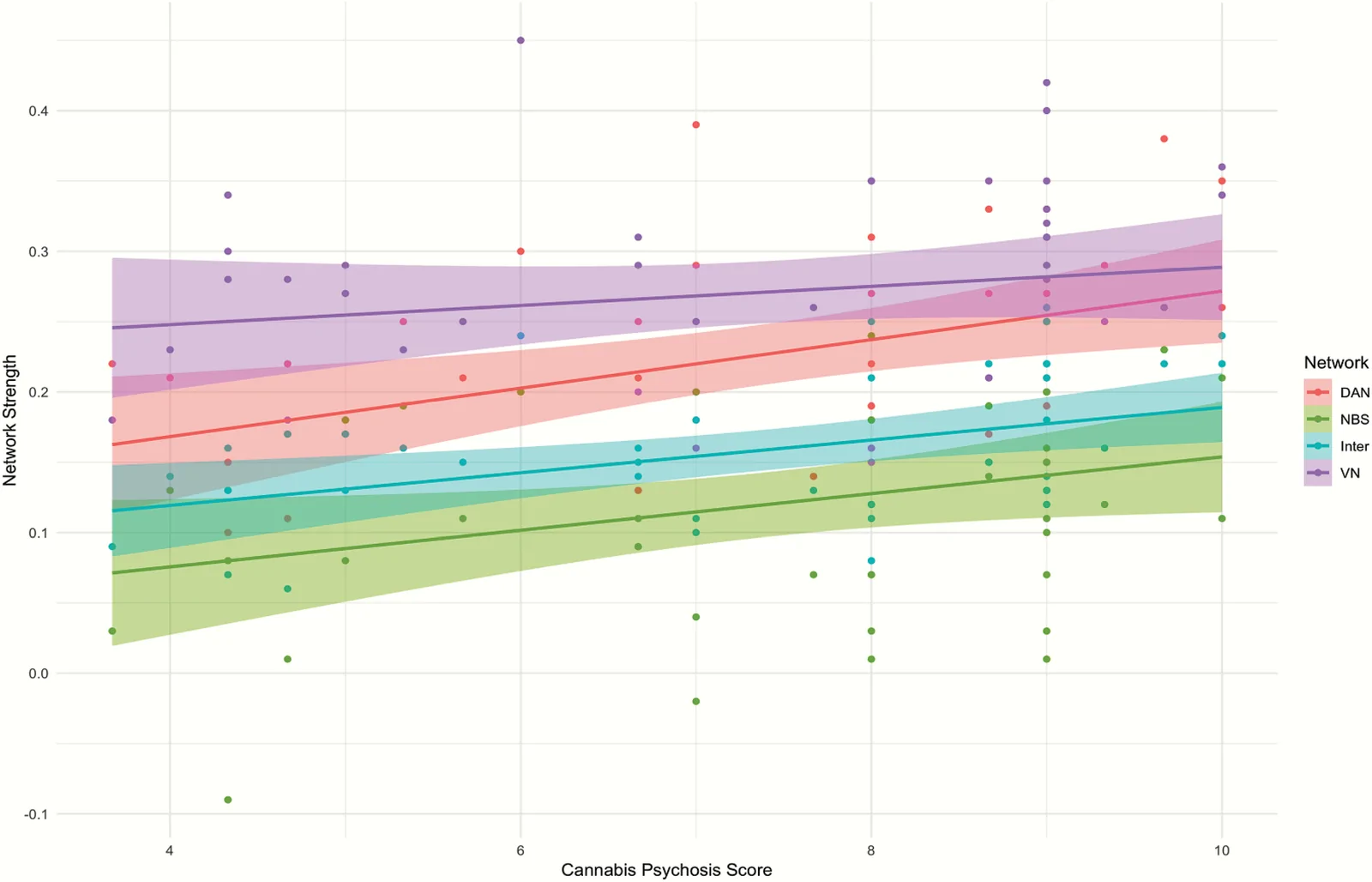
Network strength—Cannabis Psychosis Score correlation in cannabis users. DAN, Dorsal Attention Network; VN, Visual Network; Inter, Visual-Dorsal Internetwork; NBS, Network Based Statistic defined Network; Error bars represent 95% Confidence Intervals.

#### Other Clinical Parameters.

There was no correlation for network strength for all canonical networks or for the NBS network with chlorpromazine equivalents or Global Assessment of Functioning. Fagerstrom and AUDIT scores did not correlate with any network strength (*P* > .1).

#### Eye Movements.

There was no significant correlation for any network with SPEM velocity gain.

## Discussion

We set out to test differences in visual network and dorsal attention network connectivity between patients and controls with and without a history of cannabis use. We show: (1) a patient effect on the visual and visual-dorsal attention internetwork networks such that patients have hyperconnectivity compared to controls; (2) a cannabis effect on the dorsal attention network such that cannabis users have hyperconnectivity in this network compared to controls; and (3) specific evidence for hyperconnectivity in the EPC group in the dorsal attention network and visual-dorsal attention internetwork. In exploratory analysis we further demonstrate differential association in patient groups with symptomatology in the visually network and related networks and demonstrate association of parameters of cannabis use intensity with the dorsal attention network and related networks. Using the Network Based Statistic we further characterize a sparser subnetwork which demonstrates strengthened differences between groups whilst showing a similar association with symptoms and cannabis use.

To our knowledge, this is the first study to examine the visual processing and related attention networks in patients with and without a history of cannabis use. There is an extensive literature on visual processing deficits in psychotic disorders^25,45^ including abnormalities of motion contrast perception and contour detection and their neural correlates^46–49^ alongside oculomotor abnormalities.^21–24,50^ Functional MRI studies have shown abnormalities in visual and attentional areas: a visual backward masking paradigm there was reduced coupling between lateral occipital area and the superior frontal gyrus in patients with schizophrenia^51^; in a visual oddball task demonstrated disrupted connectivity between the interparietal sulcus and insula in patients with schizophrenia.^32^

Similarly, chronic cannabis use (independent of psychosis) has been shown to be associated with deficits in depth of visual processing in the perception of ambiguous visual stimuli^52^ and associated with reorganization of visual and attentional areas.^36,53^ One study has examined functional connectivity in the frontoparietal network in patients, siblings and controls, finding no significant interaction of cannabis use by group. However this may be limited due to dichotomizing cannabis into ever and never use.^54^ In contrast we recruited specifically for a history of heavy use and did not include experimental or trivial use amongst users.

Our work extends the current body of evidence to show that there is both a patient and cannabis effect in key networks involved in visual attention processing and that the association of connectivity in these networks is different in cannabis and non-cannabis using patients. Functional connectivity across a range of networks in the EPC group is associated with a history of visual hallucinations—putatively implicating these networks as markers of pathogenicity. Conversely in the EPNC group increased network strength in visual network and dorsal attention networks is significantly associated with reduced positive symptoms state measures. This may indicate an adaptive or protective response to psychosis in EPNC.

As a caveat to these inferences in a cross-sectional study we cannot definitively say that these findings represent pathogenicity or epiphenomena or a compensatory response. Further longitudinal work in chronic use and psychosis and experimental manipulation of these networks, for example through acute cannabinoid challenges or using Transcranial Magnetic Stimulation (rTMS) and transcranial Direct Current Stimulation (tCDS) may help elucidate this relationship more precisely. Δ-9-THC has been shown to disrupt hyperconnectivity of the brain reward circuit after a single dose in cannabis users^55^ whereas Default Mode Network hyperconnectivity in chronic users with psychosis is decreased after a single joint.^56^ From the findings in our study the Dorsal Attention Network may be a key network to target given the association between this network and its corrolaries with markers of cannabis use.

We did not, however, find an association between SPEM parameters and network strength indicating that these findings may be independent of group differences we previously found in smooth pursuit performance in this sample.^38^ One possible reason for this is that the SPEM findings may index a lesion in non cortical substrate such as subcortical, cerebellar and brainstem regions in eye movement processing which were not considered in this study. Alternatively the abnormality, if subsisting in cortical regions may be driven by sparse neural populations which may be beneath the limit of detection of the BOLD response. The optimal study design to investigate this difference, if detectable in fMRI, may be administration of a task based eye movement paradigm within the scanner.

We believe this study has several strengths. We derive networks using canonical networks in a well established parcellation^43^ as well as a network derived from the data (NBS). We use optimized procedures for stringent mean motion correction. Finally the patient groups (EPC and EPNC) were well matched across clinical parameters, indicating that differences were not due to a difference in illness severity, function, medication or diagnosis.

There are limitations to this study. Firstly, resting-state scans were 5 minutes which are brief but scans of this duration have been shown to reliably identify anatomic brain networks.^57,58^ Secondly since we allowed trivial cannabis use in the non-cannabis use group it is conceivable that cannabis effects could have been modulating brain activity in non-using groups. However there is of a clear demarcation between the groups in terms of cannabis use and the nonuse cannabis groups had clear evidence for trivial usage (supplementary material). Of note the Dorsal Attention Network hyperconnectivity was positively correlated with cannabis measures when examined across the entire sample. Thirdly, this study cannot fully disentangle the effect of cannabis from nicotine use in the dorsal attention network. Combined cannabis and nicotine users have been shown to have an association with increased cortical connectivity across a range of networks compared to either nicotine and cannabis users alone.^59^ However, in no network did Fagerstrom’s or AUDIT score correlate with connectivity. Our results also retain an ecological validity in a real-world sample. Another limitation is that patients we recruited were in the mild-moderate range for symptoms and functioning this may not be representative of severe patients where different brain adaptive changes may take place. This was to some extent unavoidable because patients engaged in a full study day and were recruited from a wide geographical area. Arguably the geographical representation makes the study more representative than a single site study. In supplementary analysis we did not find differences in patient status in connectivity (supplementary material) but it would be ideal to further examine these findings in a longitudinal study to determine both the effect of cannabis and medication use. Finally, for the purpose of the exploratory analysis, the Cannabis Psychosis Score is at this stage a provisional score. It has face validity and neurobiological findings we report are in line with the Severity of Dependence scale. For full validation such a score should be validated in prospective epidemiological studies and may be a future tool of interest for further research (supplementary material).

Taken together, we demonstrate both cannabis and patient effects in visual attentional networks and a pattern of hyperconnectivity in the EPC group. We show there is a distinct pattern of association between network strength and hallucinatory symptoms between patients who use and do not use cannabis. This may indicate that dysconnectivity in these networks serves different roles in the context of cannabis use.

## Funding

The EfCiP study was funded by a MRC fellowship (MR/P001408/1) to Dr Musa Sami and a minor medical grant from the Dowager Countess Eleanor Peel Trust. S.B. has received support from the National Institute for Health Research (NIHR) (NIHR Clinician Scientist Award; NIHR CS-11-001), and from the NIHR Mental Health Biomedical Research Centre at South London and Maudsley National Health Service (NHS) Foundation Trust and King’s College London. 

## Supplementary Material

sgaa018_suppl_Supplementary_Material
