# Supplementary material for: Cannabis Use Linked to Altered Functional Connectivity of the Visual Attentional Connectivity in Patients With Psychosis and Controls
Source: Schizophr Bull Open. 2020 Apr 20;1(1):sgaa018. doi: 10.1093/schizbullopen/sgaa018 (PMC13700987; doi:10.1093/schizbullopen/sgaa018)
Supplement: Supplementary file 1 [file sgaa018_suppl_supplementary_material.docx]

**Supplementary Methods:**

*Recruitment:* Patients were recruited from Early Intervention in Psychosis teams across 16 NHS Trusts in England, whereas controls were recruited from a list of interested participants and from a previous online cannabis survey run by our group (thecannabissurvey.com). Because experimental or trivial cannabis use is common in the population and our interest was in relation to heavier users which is associated with psychosis, we defined non-cannabis users as those who had used fewer than 20 times in lifetime, and in cannabis using groups (EPC, HCC) we preferentially recruited for users with a history of heavy cannabis use (see sTable 1).

*Clinical and Biological Measures:* On the study day, all participants underwent the Structured Clinical Interview for DSM-IV(1) for diagnosis, and Positive and Negative Symptoms Scale (PANSS)(2) to rate symptomatology. Function was assessed using Global Assessment of Functioning(3). Cannabis use was assessed using the Cannabis Experiences Questionnaire and the Timeline Followback Questionnaire (TFLBQ)(4). The Severity of Dependence scale was undertaken for cannabis dependence over the last 30 days(5). Tobacco use was assessed by Fagerstrom’s questionnaire and alcohol use by Alcohol Use Disorders Identification Test (AUDIT)(6). Participants underwent urine drug sampling to triangulate drug use information. Estimated full-scale IQ was calculated from a restandardised calculation of the National Adult Reading Test(7). All participants were instructed to refrain from cannabis, alcohol and caffeine use on the day of scanning. Participants were allowed to use tobacco on the day, but not smoke from one hour before resting state fMRI acquisition.

*Data acquisition:* Data were acquired using a 32-channel head coil (Nova Medical, Wilmington, MA) on a General Electric (Chicago, IL, USA) 3-Tesla system. T1 weighted images were acquired using the 3D Sagittal ADNI Go Inversion Recovery Spoiled Gradient Echo (IR-SPGR) sequence with 196 1.2mm thick slices acquired parallel to the AC-PC (anterior commissure-posterior commissure line) with an in-plane matrix size of 256 x 256 (1.05mm x 1.05mm) (TR/TI/TE 7.312ms/400ms/3.016ms, flip angle: 11°, field of view 270 mm). The field of view was placed to avoid nose wrap.

Resting state scans were acquired on the same system, using a gradient echo sequence axially depicting the blood oxygen level dependent (BOLD) signal. Participants were instructed to having keep their eyes open and fixate their gaze at a central cross. Forty-one slices were acquired with 3.0-mm thickness and 0.3mm interslice gap with in-plan matrix of 64 x 64 (3.3mm x 3.3mm) (TR/TE 2000ms/30ms, flip angle: 75°, field of view 211 mm). Total acquisition time was 5 minutes 8 seconds. The first four volumes were discarded rendering 150 volumes.

*Preprocessing and denoising:* A standard preprocessing pipeline was used consisting of realignment and normalisation to MNI space. Images were smoothed with a Gaussian kernel of 6mm full-width-half-maximum. The ARtifact detection Tools toolbox was used to account for motion and artefact detection using anatomical component based correction (aCompCor) of temporal confounds relating to head movement and physiological noise. This method models noise effects at a voxel level based on estimates derived from principal components of noise regions of interest (white matter and CSF, eroded by one voxel to minimise partial volume effects), and then removes these from the BOLD timeseries using linear regression. Six residual head motion parameters and their first order temporal derivatives were also entered as regressors into the first level model. Artifact/outlier scans (average intensity deviated more than 3 standard deviations from the mean intensity in the session, or composite head movement exceeded 1 mm from the previous image) were also regressed out. Preprocessed data were temporally bandpass filtered (0.008-0.09 Hz).

*Parcellation:* The Gordon parcellation(8) is based upon resting state boundary maps, and shows superior within parcel homogeneity when compared to other atlases, making it a valid choice for the analysis of resting state data. For each participant, a network was constructed, with each edge representing the level of functional connectivity between a pair of nodes, which was computed as the z-transformed Pearson’s correlation coefficient between their mean time-series.

*Network Based Statistic:* The hypothesis of interest (group difference) was tested at every edge across all Visual Network and Dorsal Attention edges (71 x 71 nodes, 4970 edges). This generates a single group-level network where each edge represents an F-statistic. To ensure robustness of results this network was thresholded at 100 separate F-statistic thresholds (F: 2.0 to 4.0), and the component of greatest density (i.e. greatest number of edges) identified at each threshold. Permutation testing involving the random assignment of group membership (5,000 permutations) was then employed to determine whether these identified components showed a greater density than expected by chance. The p-value for the component of interest is therefore the proportion of permutations where largest component in the ‘true’ network size is smaller than the largest component in the permuted networks. The edges of interest in networks identified as statistically significant were extracted and averaged to generate a measure of average network strength for each participant.

*Eye Movement Tracking:* Eye movement data were recorded from the right eye using an EyeLink 1000 (SR Research Ltd., Ottawa, Ontario, Canada) eye-tracker at a sampling rate of 1000 Hz. Stimulus display was on G90FB View Sonic Monitor with display area 360mm (horizontal) x 270mm (vertical), 1024 × 768 pixels, 60 Hz refresh rate. Participants were positioned with their eyes 570 mm from the monitor, on a chair adjusted for height and with the head immobilized using a comfortable chin rest with forehead restraint. The visual stimulus consisted of a white circle (0.3° diameter) on a black background with movement restricted to the horizontal plane. The smooth pursuit stimulus used a sinusoidal waveform at three target frequencies (0.2, 0.4 and 0.6 Hz) across a horizontal range of ±13°. Instructions were to follow the target as closely as possible without moving the head. Data recording was preceded by a horizontal and vertical 5-point calibration. SPEM velocity gain was calculated as eye velocity/target velocity for the middle 50% of each half-cycle, with saccades and blinks having been excluded before calculation of gain, calculated separately for each target frequency (0.2, 0.4 and 0.6 Hz).

**sTable 1 Self reported cannabis frequency by group**

|  | **EPC*** | **EPNC** | **HCC** | **HCNC** |
| --- | --- | --- | --- | --- |
| **Never use** |  | 15 (60%) |  | 11 (50%) |
| **Only once or twice** |  | 9 (36%) |  | 9 (40.9%) |
| **A few times a year** |  | 1 (4%) |  | 2 (9.1%) |
| **A few times a month** | 4 (13.8%) |  | 3 (18.8%) |  |
| **More than once a week** | 7 (24.1%) |  | 4 (25.0%) |  |
| **Daily** | 17 (58.6%) |  | 9 (56.3%) |  |

** 1 case missing as self-report history did not tally with clinician and UDS assessment*

**sTable 2: Motion Parameters between groups**

|  | **EPC** | **EPNC** | **HCC** | **HCNC** | **p-Value** |
| --- | --- | --- | --- | --- | --- |
| **n** | 29 | 25 | 16 | 22 |  |
| **Mean Motion** | 0.254 (0.195) | 0.174 (0.128) | 0.132 (0.046) | 0.202 (0.283) | 0.192 |
| **Mean Global Signal Change** | 0.926 (0.170) | 0.894 (0.128) | 0.855 (0.171) | 0.874 (0.156) | 0.476 |

**sTable 3: 2x2 ANCOVA models for all other Canonical Networks**

Patients	54

Controls	38

Cannabis Users	45

Cannabis Non-users	47

|  | **Patient Effect** | | **Cannabis Effect** | | **Patient x Cannabis Effect** | |
| --- | --- | --- | --- | --- | --- | --- |
| ***Network*** | F-test | p Value | F-test | p Value | F-test | p Value |
| *Auditory* | 0.369 | 0.545 | 0.668 | 0.416 | 0.001 | 0.975 |
| *Cingulo-opercular* | 0.899 | 0.346 | 1.014 | 0.317 | 0.004 | 0.949 |
| *Cingulo-parietal* | 0.4 | 0.529 | 0.004 | 0.952 | 0.289 | 0.592 |
| *Default Mode* | 0.552 | 0.459 | 1.408 | 0.239 | 0.047 | 0.829 |
| *Fronto-parietal* | 0.001 | 0.98 | 0.638 | 0.426 | 0.1 | 0.752 |
| *Retrospenial Temporal* | 1.174 | 0.282 | 1.347 | 0.249 | 0.712 | 0.401 |
| *Salience* | 0.406 | 0.526 | 0.005 | 0.942 | 0.209 | 0.648 |
| *Sensorimotor Hand* | 0.866 | 0.355 | 0.048 | 0.828 | 0.288 | 0.593 |
| *Sensorimotor Mouth* | 0.168 | 0.683 | 0.875 | 0.352 | 0.919 | 0.34 |
| *Ventral Attention* | 0.933 | 0.337 | 0.113 | 0.737 | 3.358 | 0.07 |
|  |  |  |  |  |  |  |
| *All tests report 2x2 ANCOVAs with patient and cannabis user as two level factor. All tests corrected for mean motion*  *Note no significant patient or cannabis effect for any network* | | | | | | |

**sFigure 1 Visual Network and Dorsal Attention Network Matrices by group:**

**(a) EPC	(b) EPNC**

**(c) HCC	(d) HCNC**

**Legend:** EPC: Patients with history of cannabis use; EPNC: Patients without history of cannabis use; HCC: Controls with history of cannabis use; HCNC: Controls without history of cannabis use. Intensity of colour signifies strength of connection (Pearson’s correlation).

**sFigure 2: NBS determination of optimal primary threshold for Sub-network**

**sFigure 3: Visual Network – Dorsal Attention Network NBS defined network matrix:**

**Legend:** Top/Left of matrices visual network; Bottom/Right of matrices Dorsal Attention Network. Colour bar indicates F-Statistic between groups. Thresholded at F-Statistic of 2.9

**sTable 4 Positive Symptoms by Group**

|  |  | ***EPC*** | | ***EPNC*** | |  |
| --- | --- | --- | --- | --- | --- | --- |
|  |  | *n* | *%ge* | *n* | *%ge* | *P-value* |
| ***Trait Measures****: Ever experienced symptoms (from SCID)* | Delusions of reference | 26 | 92.9 | 20 | 80.0 | 0.168 |
|  | Persecutory Delusions | 19 | 65.5 | 19 | 76.0 | 0.4 |
|  | Grandiose Delusions | 17 | 58.6 | 11 | 44.0 | 0.284 |
|  | Somatic Delusions | 10 | 34.5 | 5 | 20.0 | 0.236 |
|  | Other Delusions | 17 | 58.6 | 12 | 48.0 | 0.435 |
|  | Delusions of control | 11 | 37.9 | 8 | 32.0 | 0.649 |
|  | Bizarre Delusions | 19 | 65.5 | 12 | 48.0 | 0.194 |
|  | Thought disturbance | 13 | 44.8 | 11 | 44.0 | 0.951 |
|  | Auditory Hallucinations | 21 | 72.4 | 16 | 64.0 | 0.507 |
|  | Visual Hallucinations | 15 | 51.7 | 11 | 44.0 | 0.571 |
|  | Tactile Hallucinations | 13 | 44.8 | 8 | 32.0 | 0.335 |
|  | Gustatory/Olfactory Hallucinations | 8 | 27.6 | 5 | 20.0 | 0.516 |
|  |  | *Mean* | *s.d.* | *Mean* | *s.d.* | *P-value* |
| ***State Measures****: Current PANSS score* | Delusions | 2.14 | 1.41 | 2.04 | 1.49 | 0.805 |
|  | Conceptual disorganisation | 1.97 | 1.02 | 1.88 | 1.09 | 0.767 |
|  | Hallucinatory Behaviour | 1.86 | 1.27 | 2 | 1.47 | 0.713 |
|  | Excitement | 1.45 | 0.78 | 1.4 | 0.76 | 0.82 |
|  | Grandiosity | 1.52 | 0.99 | 1.56 | 1.26 | 0.889 |
|  | Suspiciousness/Persecution | 1.97 | 1.24 | 1.68 | 1.22 | 0.398 |
|  | Hostility | 1.28 | 0.59 | 1.04 | 0.20 | 0.051 |
|  | Total PANSS positive score | 12.17 | 5.40 | 11.6 | 5.45 | 0.701 |

Chi squared test for State Measures (categorical); t-test for trait measures (continuous)

**sTable 5: Positive symptoms prevalence by patient group and correlation of symptoms with networks**

| ***(a) Symptoms by Network in EPC group (n=29)*** | | **Dorsal Attention Network** | | **Visual Network** | | **Visual-Dorsal Attention Internetwork** | | **Visual-Dorsal Attention Subnetwork** | |
| --- | --- | --- | --- | --- | --- | --- | --- | --- | --- |
|  |  | rho | p-value | rho | p-value | rho | p-value | rho | p-value |
| ***Trait Measures****: Ever experienced symptoms (from SCID)* | Persecutory Delusions | 0.225 | 0.24 | -0.009 | 0.964 | 0.113 | 0.56 | 0.052 | 0.789 |
|  | Grandiose Delusions | 0.075 | 0.698 | 0.017 | 0.931 | 0.033 | 0.863 | -0.025 | 0.897 |
|  | Somatic Delusions | -0.33 | 0.081 | 0.017 | 0.929 | 0.009 | 0.964 | -0.234 | 0.222 |
|  | Other Delusions | 0.042 | 0.829 | 0.1 | 0.604 | -0.067 | 0.73 | -0.05 | 0.796 |
|  | Delusions of control | 0.255 | 0.182 | 0.136 | 0.482 | 0.272 | 0.154 | 0.399 | 0.032 |
|  | Bizarre Delusions | -0.234 | 0.222 | -0.347 | 0.065 | -0.286 | 0.132 | -0.364 | 0.052 |
|  | Thought disturbance | -0.05 | 0.798 | -0.58 | 0.765 | 0.398 | 0.033 | 0.257 | 0.179 |
|  | Auditory Hallucinations | 0.028 | 0.887 | -0.12 | 0.536 | 0.304 | 0.108 | 0.101 | 0.601 |
|  | Visual Hallucinations | 0.272 | 0.153 | 0.379 | 0.042 | 0.421 | 0.023 | 0.487 | 0.007 |
|  | Tactile Hallucinations | 0 | 1 | 0.066 | 0.733 | 0 | 1 | -0.091 | 0.638 |
|  | Gustatory/Olfactory Hallucinations | 0.175 | 0.363 | -0.46 | 0.812 | 0.341 | 0.07 | 0.258 | 0.176 |
| ***State Measures:*** *Current PANSS score* | Delusions | -0.051 | 0.792 | 0.097 | 0.616 | -0.002 | 0.99 | -0.094 | 0.629 |
|  | Conceptual disorganisation | -0.179 | 0.354 | -0.057 | 0.77 | -0.009 | 0.962 | -0.116 | 0.548 |
|  | Hallucinatory Behaviour | -0.019 | 0.921 | 0.3 | 0.113 | 0.161 | 0.404 | 0.184 | 0.34 |
|  | Excitement | -0.229 | 0.231 | -0.088 | 0.649 | -0.008 | 0.969 | -0.166 | 0.39 |
|  | Grandiosity | -0.205 | 0.286 | 0.12 | 0.534 | 0.143 | 0.459 | 0.042 | 0.83 |
|  | Suspiciousness/Persecution | 0.328 | 0.082 | 0.098 | 0.613 | 0.186 | 0.333 | 0.22 | 0.251 |
|  | Total PANSS positive score | -0.042 | 0.829 | 0.104 | 0.59 | 0.119 | 0.537 | 0.02 | 0.919 |

| ***(b) Symptoms by Network in EPNC group (n=25)*** | | **Dorsal Attention Network** | | **Visual Network** | | **Visual-Dorsal Attention Internetwork** | | **Visual-Dorsal Attention Subnetwork** | |
| --- | --- | --- | --- | --- | --- | --- | --- | --- | --- |
|  |  | rho | p-value | rho | p-value | rho | p-value | rho | p-value |
| ***Trait Measures****: Ever experienced symptoms (from SCID)* | Persecutory Delusions | 0.156 | 0.457 | 0.104 | 0.621 | 0.052 | 0.805 | 0.247 | 0.234 |
|  | Grandiose Delusions | 0.056 | 0.791 | -0.212 | 0.308 | 0.101 | 0.632 | 0 | 1 |
|  | Somatic Delusions | -0.153 | 0.467 | 0.083 | 0.693 | -0.153 | 0.467 | -0.166 | 0.427 |
|  | Other Delusions | -0.078 | 0.712 | -0.033 | 0.874 | 0.011 | 0.958 | -0.067 | 0.752 |
|  | Delusions of control | -0.119 | 0.571 | -0.036 | 0.866 | -0.321 | 0.118 | -0.143 | 0.833 |
|  | Bizarre Delusions | -0.011 | 0.958 | -0.167 | 0.426 | 0.044 | 0.833 | 0.044 | 0.496 |
|  | Thought disturbance | -0.324 | 0.114 | -0.134 | 0.523 | -0.436 | 0.029 | -0.324 | 0.114 |
|  | Auditory Hallucinations | 0.254 | 0.22 | -0.127 | 0.545 | 0.092 | 0.66 | 0.243 | 0.242 |
|  | Visual Hallucinations | 0.089 | 0.671 | -0.324 | 0.114 | -0.279 | 0.176 | -0.179 | 0.392 |
|  | Tactile Hallucinations | 0.059 | 0.778 | -0.285 | 0.167 | -0.166 | 0.426 | -0.178 | 0.426 |
|  | Gustatory/Olfactory Hallucinations | -0.125 | 0.552 | 0.069 | 0.742 | -0.111 | 0.598 | -0.14 | 0.948 |
| ***State Measures:*** *Current PANSS score* | Delusions | -0.26 | 0.209 | -0.503 | 0.01 | -0.155 | 0.46 | -0.367 | 0.071 |
|  | Conceptual disorganisation | -0.493 | 0.012 | -0.218 | 0.295 | -0.172 | 0.41 | -0.431 | 0.031 |
|  | Hallucinatory Behaviour | -0.137 | 0.514 | -0.499 | 0.011 | -0.252 | 0.225 | -0.317 | 0.123 |
|  | Excitement | -0.181 | 0.387 | -0.397 | 0.049 | -0.269 | 0.194 | -0.289 | 0.161 |
|  | Grandiosity | -0.482 | 0.015 | -0.439 | 0.028 | -0.365 | 0.073 | -0.397 | 0.049 |
|  | Suspiciousness/Persecution | -0.379 | 0.062 | -0.478 | 0.016 | -0.304 | 0.139 | -0.411 | 0.041 |
|  | Total PANSS positive score | -0.416 | 0.038 | -0.589 | 0.002 | -0.259 | 0.211 | -0.499 | 0.011 |

*Note:* Correlations for items Delusions of Reference (SCID) and Hostility (PANSS) not reported as too few case (<=3) in separate categories to report meaningful correlation

**Supplementary Analysis by patient status**

Within patients, we further undertook supplementary analysis by patient status to see if these are associated with altered connectivity in the Visual Network (VN), Dorsal Attention Network (DAN) and Visual-Dorsal Attention Internetwork (Internetwork). The three patient examined separately were (1) Schizophrenia Spectrum Disorder (i.e. schizophrenia, schizoaffective or schizophreniform disorder) versus non Schizophrenia Spectrum Disorder; (2) Affective psychotic disorder versus non affective psychotic disorder (3) Psychosis currently medicated versus Psychosis currently unmedicated.

*Note:* These findings were not compared to controls since a patient effect has already been demonstrated in the main manuscript. These findings should not affect the main results reported in the main manuscript since all patient status were matched in both patient groups (see Table 1) but are reported for interest of researchers.

*Results:*

*(1) Schizophrenia Spectrum Disorder vs Non-Schizophrenia Spectrum Disorder*

There were 41 patients with schizophrenia spectrum disorder and 13 patients with non schizophrenia psychosis. There were no significant differences between (p>0.25) the groups for any of the three networks examined.

*(2) Affective Psychotic Disorder vs Non-Affective Psychotic Disorder*

There were 35 patients with schizophrenia spectrum disorder and 19 patients with non schizophrenia psychosis. There were no significant differences between groups for any of the three networks examined (DAN p=0.72, VN p=0.951, Internetwork p=0.277).

*(3) Medicated psychosis vs Unmedicated psychosis*

There were 36 patients with schizophrenia spectrum disorder and 18 patients with non schizophrenia psychosis. There were no significant differences between (p>0.16) the groups for any of the three networks examined.

Interpretation: We conclude from our results we cannot find evidence that these patient status factors are associated with altered functional connectivity within patients. These results should be interpreted with caution as sample sizes may be underpowered to detect a true difference.

**Calculation of the Cannabis Psychosis Score (CPS):**

The Cannabis Psychosis Score (CPS) has been calculated in order to determine a provisional score for use-behaviours established to be associated with psychosis risk and adverse outcome in psychotic disorders. Contemporary instruments fail to capture these use behaviours in a single score. This is problematic because there is a heterogeneity of use behaviours associated with psychosis which are not captured in a single instrument. For example, an individual may have commenced cannabis use early in adolescence and developed a dependence on cannabis but now stopped, whereas another individual may be using high-potency cannabis such as ‘skunk’ persistently and heavily but commenced later in life and not meet the criteria for cannabis abuse. Both scenarios are well established to be associated with adverse psychosis outcomes, but would fail to be captured in a single metric. Furthermore evidence indicates that adverse use-behaviours are synergistic(9–11). Conversely an individual may have met the criteria for substance abuse due to legal problems but have used infrequently and low-potency, and another individual may have used high potency cannabis at an early age but only a few times. These are lower risk behaviours but would still score high if looked at from a single domain.

For the CPS we hence scored use behaviours across five domains known to be associated with psychosis: (i) heaviness of use(12, 13); (ii) early first use(14, 15); (iii) cannabis abuse or dependence(16, 17); (iv) persistence of use(18–20); and (v) potency of use(11, 21).

In the EfCiP study the CPS was calculated from standard research and clinical instruments assessing use (TimeLine Followback Questionnaire(4), Cannabis Experiences Questionnaire modified version(22), SCID for DSM IV substance abuse module(1), and Urine Drug Sampling. We excluded one individual where there was contradiction between UDS and self reported consumption of cannabis. The following score was calculated:

| **Domain and Instrument** | *Scoring Instructions* | *Score* |  |
| --- | --- | --- | --- |
| **Persistence**  *Instrument:*  *UDS/CEQ/TFLB* | - Never used - Ever used - Urine Drug Sample positive *or* self reported use in last 30 days (if UDS unavailable) | 0  1  2 | /2 |
| **Abuse**  *Instrument:*  *SCID* | - No lifetime diagnosis of cannabis abuse - Lifetime history of cannabis abuse - Lifetime history of cannabis dependence | 0  1  2 | /2 |
| **Age of first use**  *Instrument:*  *CEQ/SCID* | - Never used - 17 and above - 16 and below | 0  1  2 | /2 |
| **Money spent /week on cannabis**  *Instrument:*  *CEQ* | - Never or experimental (less than 5 times) - Upto to £2.50 - £2.50 - £5 - £6 - £10 - £10 to £15 - £16 - £20 - Above £20 | 0  1 ÷ 3 = 0.33..  2 ÷ 3 = 0.66..  3 ÷ 3 = 1  4 ÷ 3= 1.33..  5 ÷ 3 = 1.66..  6 ÷ 3 = 2 | /2 |
| **Potency score**  *Instrument:*  *CEQ* | - No use or experimental - Low potency* - High potency* such as ‘skunk’*   **If unknown if participant indicates has strong effect score as high potency, if participant does not indicate this score as low potency* | 0  1  2 | /2 |
|  |  | **TOTAL** | **/10** |

**Properties of the CPS score in the EfCiP sample:**

*1 Group Differences:*

Group differences of the CPS score are, as expected higher in users compared to non-using groups and distinguish between the two:

*

*

*

*

*2 Correlation with other cannabis using measure:*

The CPS score moderately to strongly positively correlated with the Severity of Dependence Scale (SDS) for cannabis which indexes dependence features over the previous 30 days. Consequently both SDS and CPS measure different constructs. However, the SDS is less sensitive to previous cannabis use with discernable floor effects and only scored above 0 in 17/92 of the current sample, whereas the CPS score is more evenly distributed.

*3 Association with clinical measures in the early psychosis group with a history of cannabis use:*

The CPS score was moderately inversely correlated with GAF in the EPC group, with a higher score indicating worse functioning:

*4 Neuroimaging results:*

As shown in the main manuscript the CPS score correlated with Dorsal Attention connectivity (rho=0.281, p=0.007). This is consistent with other cannabis measures: Dorsal Attention network strength is noted to be highest in patients with a history of cannabis use, and correlated with the cannabis Severity of Dependence Scale.

Taken together the CPS (i) has primary face validity; (ii) is associated with other cannabis using measures; (iii) is associated with worse functioning in patients with psychosis with a history of cannabis use; (iv) is associated with cannabis related neuroimaging results in our sample; and further (v) can be easily calculated from standardised instruments. Further confirmation and refinement of the score needs to be undertaken in prospective epidemiological samples.

**References:**

1. First MB, Spitzer RL, Gibbon M, Williams JBW, Davies M, Borus J, *et al.* (1995): *The Structured Clinical Interview for DSM-IV Axis I Disorders-Patient Edition*. *Biometrics Res Dep*. . doi: 10.1521/pedi.1995.9.2.92.

2. Kay SR, Fiszbein A, Opler LA (1987): The Positive and Negative Syndrome Scale (PANSS) for Schizophrenia. *Schizophr Bull*. 13: 261–276.

3. Association AP (23AD): *DSM-IV: Diagnostic and Statistical Manual of Mental Disorders - fourth edition*. *Diagnostic Stat Man Ment Disord*. . doi: 10.1001/jama.1994.03520100096046.

4. Sobell LC, Sobell MB (1996): Timeline Followback user’s guide: A calendar method for assessing alcohol and drug use. *Toronto Addict Res Found*. .

5. Gossop M, Darke S, Griffiths P, Hando J, Powis B, Hall W, Strang J (1995): The severity of dependence scale (SDS): Psychometric properties of the SDS in English and Australian samples of heroin, cocaine and amphetamine users. *Addiction*. 90: 607–614.

6. Saunders JB, Aasland OG, Babor TF, de la Fuente JR, Grant M (1993): *Creation of the Alcohol Use Disorders Identification Test (AUDIT): WHO Collaborative Project Addiction*. *Addiction*. (Vol. 88). Retrieved from http://www.ncbi.nlm.nih.gov/pubmed/8329970.

7. Bright P, Hale E, Gooch VJ, Myhill T, van der Linde I (2018): The National Adult Reading Test: restandardisation against the Wechsler Adult Intelligence Scale-Fourth edition. *Neuropsychol Rehabil*. 28: 1019–1027.

8. Gordon EM, Laumann TO, Adeyemo B, Huckins JF, Kelley WM, Petersen SE (2016): Generation and Evaluation of a Cortical Area Parcellation from Resting-State Correlations. *Cereb Cortex*. 26: 288–303.

9. Di Forti M, Marconi A, Carra E, Fraietta S, Trotta A, Bonomo M, *et al.* (2015): Proportion of patients in south London with first-episode psychosis attributable to use of high potency cannabis: A case-control study. *The Lancet Psychiatry*. 2: 233–238.

10. Di Forti M, Quattrone D, Freeman TP, Tripoli G, Gayer-Anderson C, Quigley H, *et al.* (2019): The contribution of cannabis use to variation in the incidence of psychotic disorder across Europe (EU-GEI): a multicentre case-control study. *The Lancet Psychiatry*. 6: 427–436.

11. Schoeler T, Petros N, Di Forti M, Klamerus E, Foglia E, Ajnakina O, *et al.* (2016): Effects of continuation, frequency and type of cannabis use on relapse in the first two years following onset of psychosis - an observational study. *Lancet Psychiatry*. 366: 1–7.

12. Di Forti M, Sallis H, Allegri F, Trotta A, Ferraro L, Stilo SA, *et al.* (2014): Daily use, especially of high-potency cannabis, drives the earlier onset of psychosis in cannabis users. *Schizophr Bull*. 40: 1509–1517.

13. Marconi A, Di Forti M, Lewis CM, Murray RM, Vassos E (2016): Meta-analysis of the Association Between the Level of Cannabis Use and Risk of Psychosis. *Schizophr Bull*. 42: 1262–1269.

14. Bagot KS, Milin R, Kaminer Y (2015): Adolescent Initiation of Cannabis Use and Early-Onset Psychosis. *Subst Abus*. 36.

15. Malone DT, Hill MN, Rubino T (2010): Adolescent cannabis use and psychosis: Epidemiology and neurodevelopmental models. *Br J Pharmacol*. 160.

16. Wisdom JP, Manuel JI (2011): Prevalence of substance use in people with first-episode Psychosis. *J Dual Diagn*. 7: 39–49.

17. Fergusson DM, Horwood LJ, Swain-Campbell NR (2003): Cannabis dependence and psychotic symptoms in young people. *Psychol Med*. 33: 15–21.

18. Schoeler T, Monk A, Sami MB, Klamerus E, Foglia E, Brown R, *et al.* (2016): Continued versus discontinued cannabis use in patients with psychosis: A systematic review and meta-analysis. *The Lancet Psychiatry*. 3: 215–225.

19. Kuepper R, van Os J, Lieb R, Wittchen H-U, Höfler M, Henquet C (2011): Continued cannabis use and risk of incidence and persistence of psychotic symptoms: 10 year follow-up cohort study. *BMJ*. 342: d738.

20. González-Pinto A, Alberich S, Barbeito S, Gutierrez M, Vega P, Ibáñez B, *et al.* (2011): Cannabis and first-episode psychosis: Different long-term outcomes depending on continued or discontinued use. *Schizophr Bull*. 37: 631–639.

21. Di Forti M, Morgan C, Dazzan P, Pariante C, Mondelli V, Marques TR, *et al.* (2009): High-potency cannabis and the risk of psychosis. *Br J Psychiatry*. 195: 488–491.

22. Schoeler T, Petros N, Di Forti M, Pingault J-B, Klamerus E, Foglia E, *et al.* (2016): Association Between Continued Cannabis Use and Risk of Relapse in First-Episode Psychosis. *JAMA Psychiatry*. 35: 557–574.
